# Intramolecular Regulation of Phosphorylation Status of the Circadian Clock Protein KaiC

**DOI:** 10.1371/journal.pone.0007509

**Published:** 2009-11-25

**Authors:** Yao Xu, Tetsuya Mori, Ximing Qin, Heping Yan, Martin Egli, Carl Hirschie Johnson

**Affiliations:** 1 Department of Biological Sciences, Vanderbilt University, Nashville, Tennessee, United States of America; 2 Department of Biochemistry, Vanderbilt University Medical Center, Nashville, Tennessee, United States of America; Massachusetts Institute of Technology, United States of America

## Abstract

**Background:**

KaiC, a central clock protein in cyanobacteria, undergoes circadian oscillations between hypophosphorylated and hyperphosphorylated forms *in vivo* and *in vitro*. Structural analyses of KaiC crystals have identified threonine and serine residues in KaiC at three residues (T426, S431, and T432) as potential sites at which KaiC is phosphorylated; mutation of any of these three sites to alanine abolishes rhythmicity, revealing an essential clock role for each residue separately and for KaiC phosphorylation in general. Mass spectrometry studies confirmed that the S431 and T432 residues are key phosphorylation sites, however, the role of the threonine residue at position 426 was not clear from the mass spectrometry measurements.

**Methodology and Principal Findings:**

Mutational approaches and biochemical analyses of KaiC support a key role for T426 in control of the KaiC phosphorylation status *in vivo* and *in vitro* and demonstrates that alternative amino acids at residue 426 dramatically affect KaiC's properties *in vivo* and *in vitro*, especially genetic dominance/recessive relationships, KaiC dephosphorylation, and the formation of complexes of KaiC with KaiA and KaiB. These mutations alter key circadian properties, including period, amplitude, robustness, and temperature compensation. Crystallographic analyses indicate that the T426 site is phosphorylatible under some conditions, and *in vitro* phosphorylation assays of KaiC demonstrate labile phosphorylation of KaiC when the primary S431 and T432 sites are blocked.

**Conclusions and Significance:**

T426 is a crucial site that regulates KaiC phosphorylation status *in vivo* and *in vitro* and these studies underscore the importance of KaiC phosphorylation status in the essential cyanobacterial circadian functions. The regulatory roles of these phosphorylation sites–including T426–within KaiC enhance our understanding of the molecular mechanism underlying circadian rhythm generation in cyanobacteria.

## Introduction

Circadian clocks are self-sustained intracellular oscillators that regulate daily rhythms of sleep/waking, metabolic activity, gene expression, and many other biological processes. Cyanobacteria are the simplest organisms known to exhibit circadian rhythms [1]. In the cyanobacterium, *Synechococcus elongatus* PCC 7942, a cluster of three clock genes, named *kaiA*, *kaiB*, and *kaiC*, encodes essential components of the circadian clock [2]; if any of these genes is knocked out, circadian rhythmicity is abolished in *S. elongatus* cells. The Kai proteins have been crystallized and their three-dimensional structures have been determined [3]. Remarkably, a circadian oscillation of KaiC phosphorylation status can be reconstituted *in vitro* with just the three purified Kai proteins [4]. The relationship of this *in vitro* oscillator to the entire circadian system *in vivo* is not defined, but it is clear that the rhythm of KaiC phosphorylation is able to keep circadian time independently of transcription and translation processes *in vivo* [3], [5], suggesting that the KaiABC oscillator is necessary and sufficient as a core oscillator for circadian rhythmicity in cyanobacteria. The circadian pacemaker in *S. elongatus* choreographs rhythmic patterns of global gene expression, chromosomal compaction, and the supercoiling status of DNA *in vivo* [6]–[8]. One hypothesis (the “Oscilloid Model”) suggests that the regulation of gene expression is mediated through the clock regulating chromosomal topology that in turn regulates promoter activities by torsion-sensitive transcription [8], [9], while an alternative (and not mutually exclusive) model suggests that the KaiABC oscillator regulates the activity of transcriptional factors such as RpaA that regulate global changes of gene expression [10].

The Kai proteins interact with each other and influence each other's activity [11]–[16]. These interactions lead to the formation of large complexes with KaiC as the core. These complexes mediate the KaiC oscillation between hypophosphorylated and hyperphosphorylated forms *in vivo* and *in vitro* [4], [5], [17]–[20]. In the *in vitro* system, Kai protein complexes assemble and disassemble dynamically over the KaiC phosphorylation cycle [14], [16]. In addition, there is an exchange of monomers among KaiC hexamers that occurs during the KaiC dephosphorylation phase [16], [21]. KaiC autophosphorylation is stimulated by KaiA, whereas KaiB antagonizes the effects of KaiA on KaiC autophosphorylation [17], [22], [23]. On the other hand, dephosphorylation of KaiC is inhibited by KaiA, and this effect of KaiA is also antagonized by KaiB [20]. Therefore, KaiA both stimulates KaiC autophosphorylation *and* inhibits its dephosphorylation; KaiB antagonizes these actions of KaiA.

The crystal structure of the KaiC hexamer revealed ATP binding, insights into KaiC inter-subunit organization, and a scaffold for Kai-protein complex formation [24]. The KaiC structure also shed light on the mechanism of rhythmic phosphorylation of KaiC by identifying three potential phosphorylation sites at threonine and serine residues in KaiC at residues T426, S431, and T432 [25]. An important role for these residues was shown by the loss of rhythmicity *in vivo* when any of these residues were mutated to alanine (i.e., T426A, S431A, or T432A; 25). Based on mass spectrometry studies, Nishiwaki and coworkers also showed that S431 and T432 are key phosphorylation sites in KaiC [19]. Moreover, phosphorylation of S431 and T432 occurs in an ordered sequence over the cycle of the *in vitro* KaiABC oscillator [26], [27]. These phosphorylation events are likely to mediate conformational changes in KaiC that allow interaction with KaiB and subsequent steps in the molecular cycle [3].

However, the role of the T426 residue in the phosphorylation/conformation cycle is unclear. The crystal structure of KaiC revealed that T426 and S431 face each other across a looped region and that an extra electron density that can be attributed to the presence of a phosphate exists between T426 and S431 in some of the subunits [25]. Moreover, mutation of T426 to a non-phosphorylatible alanine abolishes rhythmicity. We therefore speculated that this putative phosphate group shuttles between these two residues, i.e. that S431 and T426 share a phosphate. On the other hand, the mass spectrometry studies did not find T426 to be phosphorylated [26], [27]. In the present study, we demonstrate that T426 is a crucial site that regulates KaiC phosphorylation status and is essential for cyanobacterial circadian functions, including period, amplitude, robustness, and temperature compensation. *In vivo* co-expression of wild-type KaiC with KaiC mutated at T426 establishes dominant-negative and semi-dominant genetic relationships and alters temperature compensation. *In vitro*, asparagine/glutamate vs. alanine mutations at 426 differentially affect KaiC's biochemical properties–especially the kinetics of KaiC dephosphorylation and the association of KaiC with KaiA and KaiB into complexes. We find that KaiC undergoes an unstable phosphorylation when the S431 and T432 sites are blocked, and crystallographic analyses described in the accompanying paper [28] indicate that this labile phosphorylation status could be at the T426 site. The regulatory roles of these phosphorylation sites (S431, T432, *and* T426) within KaiC provide further evidence towards understanding the molecular mechanism responsible for circadian rhythm generation in cyanobacteria.

## Materials and Methods

### Nomenclature of KaiC mutants

In this paper, KaiC variants are sequentially shown as a superscript format in order of sites 426, 431, and 432 where the wild-type residue is in upper case and mutated residues are shown in lower case, i.e. KaiC^xyz^, where x  =  residue at position 426, y  =  residue at position 431, and z  =  residue at position 432. For example, KaiC^TST^ is for the wild-type KaiC^T426/S431/T432^ (i.e. KaiC^WT^), KaiC^aST^ for KaiC^T426A/S431/T432^, KaiC^Tae^ for KaiC^T426/S431A/T432E^, etc.

### Generation of KaiC mutants at phosphorylation sites

Mutation of KaiC at 426, 431, and 432 was performed by site-directed mutagenesis based on template plasmids harboring either wild-type *kaiABC* or *kaiC DNA*. Briefly, the mutated *kaiC* strand DNA was synthesized using 12∼18 thermal cycles with pfuUltra® High-fidelity DNA polymerase (Stratagene, San Diego, CA) and primers containing the desired mutations. After the nonmutated parental plasmids were digested at 30°C for 1 h with *Dpn*I (New England Biolabs, Beverly, MA), the circular, nicked mutant dsDNA was transformed into *E. coli* DH5a strain and the constructs were confirmed by DNA sequencing.

### Cyanobacterial Strains

**Figure 1:**
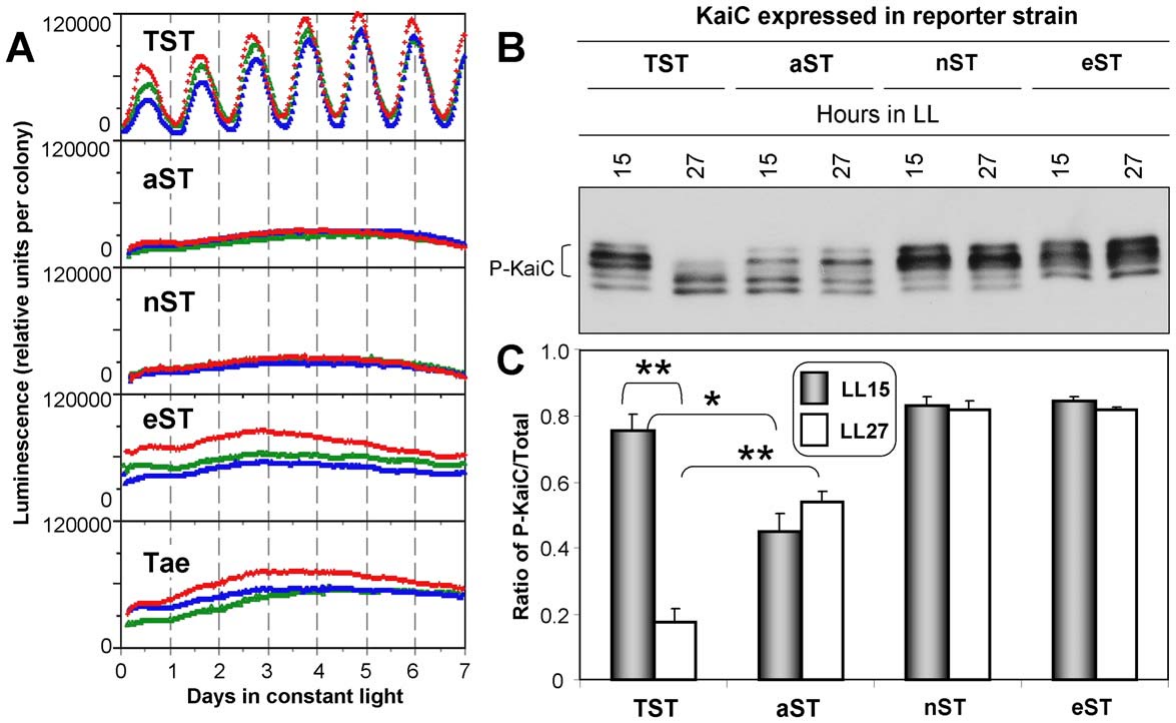
Mutations at residue 426 abolish circadian rhythms of luminescence *in vivo*. This figure summarizes the effects of expressing a single copy of KaiC (either wild-type or mutated version) in cyanobacterial cells. (***A***) Effect of single mutations at T426 and double mutations at S431/T432 on the *kaiBC*p-driven luminescence rhythm and expression levels. The luminescence rhythms were obtained from strains expressing wild-type KaiC (TST) or mutant KaiCs (aST, nST, eST, or Tae) in constant light (LL). Three replicates for each case are shown in different colors. (***B***) T426 mutations alter KaiC phosphorylation profiles and abolish KaiC phosphorylation rhythms. The cultures expressing wild type or mutant KaiCs were harvested at 15 h or 27 h in LL and extracts were analyzed by immunoblot. P-KaiC denotes hyperphosphorylated KaiC bands. (***C***) Ratios (mean±SD) of the P-KaiC:total KaiC ratio from densitometry of data in panel *B*. *  =  *p*<0.05; **  =  *p*<0.01 (by Student's t-test).

For expression of a single copy of *kaiC* in cyanobacteria with a *kaiBC*p::luxAB reporter (Figs. 1 and 3C), the wild-type *kaiABC* cluster was replaced by a *kaiABC* cluster containing wild-type or mutant *kaiC* and a spectinomycin selection marker.

For co-expression of KaiC in cyanobacteria, the *trc*p::KaiC construct (with or without mutations in *kaiC*) was integrated into the NS II region of a wild-type strain with a *psbAI*p::luxAB reporter in NS I. The *kaiABC*-null strain was created by replacement of the *kaiABC* DNA region with a kanamycin resistance gene [2]. Descriptions of these strains are listed in Table S1. The clock-controlled activation of the *kaiBC*p::luxAB reporter was monitored by real-time measurement of luminescence as described previously [20].

### Immunoblot assays for KaiC phosphorylation

After two LD 12:12 cycles, the cultures were released to constant light (LL). The cells were harvested at different phases, and the protein extracts were subjected to SDS-PAGE separation and immunoblot analyses with a mouse polyclonal antibody directed against KaiC as described previously [20], [25]. For comparison of the soluble and insoluble KaiC fractions in cyanobacterial cells after a 6 h induction with IPTG (0.1 mM), cells were harvested and sonicated in a SDS-free buffer (50 mM HEPES pH 7.4, 130 mM KCl, 5% glycerol, 2.5 mM EDTA, 10 µg/ml aprotinin, 10 µg/ml leupeptin, 2 µg/ml pepstatin and 5 mM phenylmethylsulfonyl fluoride) and microcentrifuged for 3 min. The supernatant was collected and the pellet was washed three times in the same buffer. The pellet (insoluble fraction) and supernatant (soluble fraction) were then separately prepared for SDS-PAGE and immunoblotting.

### Computation of Q_10_ values

Cyanobacterial growth, genetic transformation, and luminescence recordings were performed as previously described [20], [25]. Periods and phases of luminescence rhythms were analyzed with ChronoAnalysis II, version 10.1 (courtesy of Dr. Till Roenneberg). Temperature compensation was quantified by calculation of Q_10_ with the following equation: Q_10_  =  [(1/t_2_)/(1/t_1_)]^10/(T2 – T1)^; where: t_1_  =  period at the lower temperature (T1) and t_2_  =  period at the higher temperature (T2; in this study, T1 = 24°C and T2 = 30°C).

### ^32^P-labeled KaiC phosphorylation *in vitro*

Purified proteins (200 ng/µl) of wild-type KaiC^TST^ or mutant KaiCs (aST, Tae, aaa, or aae) were incubated at 30°C in Reaction Buffer (RB; 150 mM NaCl, 5 mM MgCl_2_, 1 mM ATP, 0.5 mM EDTA, 20 mM Tris-HCl, pH 8.0] containing 0.4 µCi/µl [γ-^32^P]ATP. At each time point, 10 µl aliquots were taken, mixed with 10 µl of 2X SDS-PAGE loading buffer, and stored at −20°C. Before SDS-PAGE, the samples were either (i) not heated above room temperature, (ii) heated to 72°C for 10 min, or (iii) heated to 100°C for 10 min as indicated. Following SDS-PAGE, gels were washed and fixed in 20% MeOH and 7% HOAc overnight, stained with colloidal Coomassie Brilliant Blue (CBB) overnight, destained in MQ-water for several hours, and dried at 68°C for 4 hours. The dried gels were exposed to a phosphorimager screen, which was scanned by a Typhoon Imager (GE Healthcare).

### KaiB/KaiA antagonism of KaiC phosphorylation/dephosphorylation *in vitro*

Purified wild-type or mutant KaiC proteins (100 ng/µl) were incubated at 30°C in RB with or without 25 ng/µl of the purified KaiA and/or KaiB at 30°C in a time course up to 24 h. The *in vitro* KaiC phosphorylation/dephosphorylation patterns were examined by SDS-PAGE analysis.

### Analysis of stable KaiBC and KaiABC complexes

To analyze the formation of stable complexes between KaiB and KaiC, KaiB (50 ng/µl) and KaiC (200 ng/µl) were mixed and incubated at 30°C in a volume of 100 µl RB. 16 µl aliquots of each mixture were collected at each time point and mixed with 5X non-denaturing buffer (0.312 M Tris-HCl, pH 6.8, 0.05% CBB, 50% glycerol) and frozen immediately at −80°C. Native gel electrophoresis (7.5% polyacrylamide) was performed at 4°C to separate protein complexes. Gels were stained with colloidal CBB, and gel images were digitally captured with the Bio-Rad Gel Doc XR system (Bio-Rad, Hercules, CA).

## Results and Discussion

### Mutations at T426 that prevent phosphorylation abolish rhythmicity *in vivo*

We previously reported that mutation of T426 to the non-phosphorylatible residue alanine abolished rhythmicity [25]. We confirmed in this study that replacement of wild-type KaiC (i.e. KaiC^TST^) with KaiC^aST^ not only abolishes rhythmicity, but also constitutively represses reporter activity *in vivo* (Fig. 1A). If T426 is replaced with glutamate that could serve as a mimic of a phosphorylated residue (KaiC^eST^), the cells are also arhythmic. Therefore, neither constitutive activation at T426 (KaiC^eST^) nor constitutive inactivation (KaiC^aST^) can support rhythmicity. Kitayama and co-workers reported that a KaiC with glutamate at both 431 and 432 sites (KaiC^Tee^) was able to support rhythmicity, albeit with a damped, long period rhythm [29], implying that phosphorylatibility at T426 could be sufficient to allow rhythmicity if both 431 and 432 are constitutively activated by glutamate residues. To test if phosphorylatibility at T426 is sufficient to allow rhythmicity when 431 cannot be phosphorylated, we examined a double mutant KaiC^Tae^, where 432 is activated by glutamate, but 431 is a non-phophorylatible and non-activated alanine. From the crystal structure, T426 can be phosphorylated in KaiC^Tae^ *in vitro* ([28], and see below). However, KaiC^Tae^ cannot support rhythmicity *in vivo* (Fig. 1A). Because KaiC^Tee^ allows rhythmicity *in vivo* [29] but KaiC^Tae^ does not, clearly 431 needs to be activated by phosphorylation or as a glutamate. It is likely that 431 must be activated by phosphorylation or as a glutamate in order for KaiB to bind KaiC and initiate the dephosphorylation cycle [3], [26], [27].

The electron density between S431 and T426 in the crystal structure of KaiC^TST^ [24], [25] is placed such that the center of the electron density (the putative phosphorylation) is 1.6 Å from the γ-oxygen of S431, which is appropriate for a covalent bond between S431 and the putative phosphate. The center of this electron density is 4.5 Å from the γ-oxygen of T426, which also places the phosphate within hydrogen bonding distance from T426. Therefore, to test if the arhythmicity of KaiC^aST^ is due to the inability of (i) A426 to form a hydrogen bond to the phosphate group attached to S431, or (ii) A426 to be phosphorylated (and therefore that T426 could be a *bona fide* phosphorylation site), we mutated the 426 residue to asparagine, which can accept or donate hydrogen bonds (also, asparagine is roughly isosteric with threonine), but which cannot be phosphorylated. Therefore, the predictions are: (i) if the function of 426 is to interact with the phosphorylated residue S431 via a hydrogen bond, then KaiC^nST^ should be rhythmic, whereas (ii) if the 426 site needs to be phosphorylatible, then KaiC^nST^ will be dysfunctional as well. Fig. 1A shows that KaiC^nST^ is also arhythmic *in vivo*; the results with KaiC^aST^, KaiC^eST^, and KaiC^nST^ support the conclusion that regulation of phosphorylation at 426 is crucial and that the function of threonine at 426 is not merely to hydrogen bond with a phosphate attached to S431.

### Phosphorylation patterns of T426 mutants *in vivo*

When extracts of *S. elongatus* cells are immunoblotted with an antibody to KaiC, the phosphorylation states of KaiC can be visualized by mobility shifts that show hyperphosphorylation during the subjective night (hours 12–24 in LL for KaiC^TST^) and hypophosphorylation in the subjective day (hours 24–36 for KaiC^TST^) [3], [26], [27]. A 54 h time course of phosphorylation patterns *in vivo* for KaiC^TST^ *vs.* mutant KaiCs reveals that none of the KaiC mutations at 426, 431, or 432 (including KaiC^aST^) can sustain a rhythm of KaiC phosphorylation (Fig. S1). While none of the KaiC mutations related to residue 426 show rhythms of phosphorylation, they do affect the overall phosphorylation patterns *in vivo* (Figs. 1B, 1C). KaiC^nST^ and KaiC^eST^ are relatively hyperphosphorylated, suggesting that asparagine or glutamate at 426 might inhibit dephosphorylation at S431 and T432. Note the complete absence of the lowest KaiC band (i.e., non-phosphorylated KaiC) in KaiC^eST^. On the other hand, KaiC^Tae^ is relatively hypophosphorylated, indicating a weak ability to stably phosphorylate if the key residue 431 is not phosphorylatible (see below). The pattern depicted for KaiC^aST^ in Fig. 1B is particularly interesting. First, KaiC^aST^ clearly shows five bands (implying at least five phospho-states of KaiC *in vivo*), whereas usually only four bands are observed over the circadian cycle *in vitro* [26], [27]. Second, the KaiC^aST^ pattern shows both hyperphospho-states (upper bands) and hypophospho-states (lower bands) but the middle band is missing or greatly reduced in KaiC^aST^. It is tempting to speculate that this middle band (third from the top) indicates a phospho-state in which T426 is phosphorylated and which has been captured in the crystal structure of KaiC^Tae^ [28].

### Co-expression of 426 mutants with wild-type KaiC *in vivo*

**Figure 2 pone-0007509-g002:**
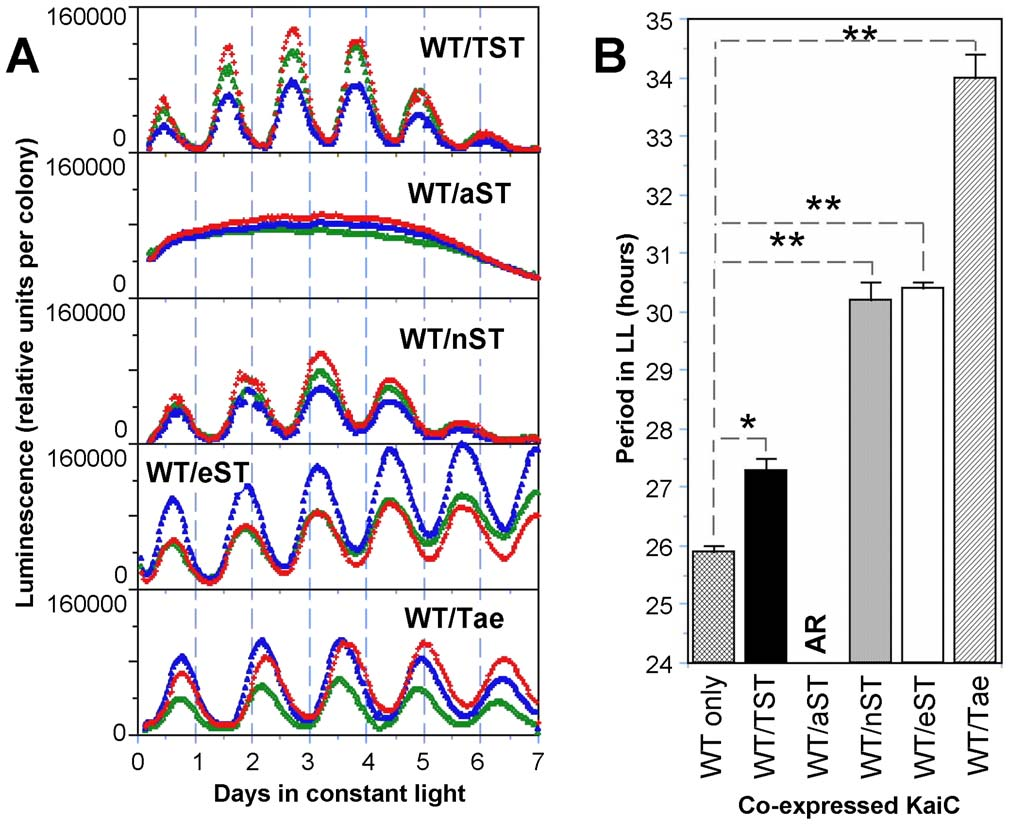
Co-expression reveals genetic relationships of KaiC^TST^ with mutant versions of KaiC *in vivo*. This figure summarizes the effects of co-expressing KaiC^TST^ (WT) with another KaiC (either wild-type or mutated version) under the control of *trc*p in the absence of IPTG induction. (***A***) Luminescence reporter strain with the endogenous *kaiABC* cluster and also expressing an additional copy of (i) wild-type KaiC (WT/TST), (ii) T426 single mutant KaiCs (WT/aST, WT/nST, or WT/eST), or (iii) S431/T432 double mutant KaiC (WT/Tae). Three replicates for each combination are shown in different colors. (***B***) Comparison of periods of the luminescence rhythms in LL at 30°C between wild-type strain (WT only) and various KaiC-coexpressing strains. Error bars are standard deviation (SD). *, *p*<0.05; **, *p*<0.01 (by Student's t-test). AR  =  arhythmia.

Another way to test the significance of the T426 site is to co-express the KaiC mutants at this site with KaiC^TST^ *in vivo* and assess whether the mutant KaiCs affect or disrupt the rhythmicity imparted by the wild-type KaiC. All versions of KaiC (KaiC^TST^ and mutant versions) show similar levels of expression of soluble KaiC when expressed from an ectopic site under the control of a *trc* promoter (*trc*p; 20) (Fig. S2). When wild-type KaiC (KaiC^TST^) is expressed in a wild-type strain in which the endogenous *kaiABC* cluster is present, rhythmicity is preserved with a slightly longer period from ∼26.0 h to ∼27.2 h at 30°C (Fig. 2). When the KaiC^nST^, KaiC^eST^, and Kai^Tae^ mutants are co-expressed with KaiC^TST^ under the same conditions, the period is lengthened very significantly from ∼26 h to ∼30–34 h (p<0.01; Fig. 2B). Not only the period, but also temperature compensation is affected by the co-expression of these mutants because the Q_10_ increases from 1.01 to ∼1.3 (Table 1). This result means that co-expression of these 426 site mutants alters the properties of the oscillating system, but does not prevent KaiC^TST^ from maintaining the rhythm. On the other hand, when KaiC^aST^ is co-expressed with KaiC^TST^ under the same conditions, the T426A mutation always exerts a dominant negative effect on the luminescence rhythms (Fig. 2A) independent of the level of expression of KaiC^aST^. Therefore, the KaiC^aST^ mutant interferes with the function of the endogenous KaiC^TST^.

**Table 1:** Co-expression of mutant KaiCs alters temperature compensation of the clock *in vivo*.

Version of KaiC co-expressed *in vivo* with wild type KaiC	Period at 24°C (mean ± SD)	Period at 30°C (mean ± SD)	Q_10_
KaiC^TST^ (WT)	27.6±0.1	27.5±0.1	1.01
KaiC^aST^ (T426A)	Arhythmic	Arhythmic	---
KaiC^nST^ (T426N)	36.4±0.6	30.2±0.3	1.36
KaiC^eST^ (T426E)	35.7±0.3	30.4±0.2	1.31
KaiC^Tae^ (S431A/T432E)	39.3±0.6	34.0±0.3	1.27

**Figure 3:**
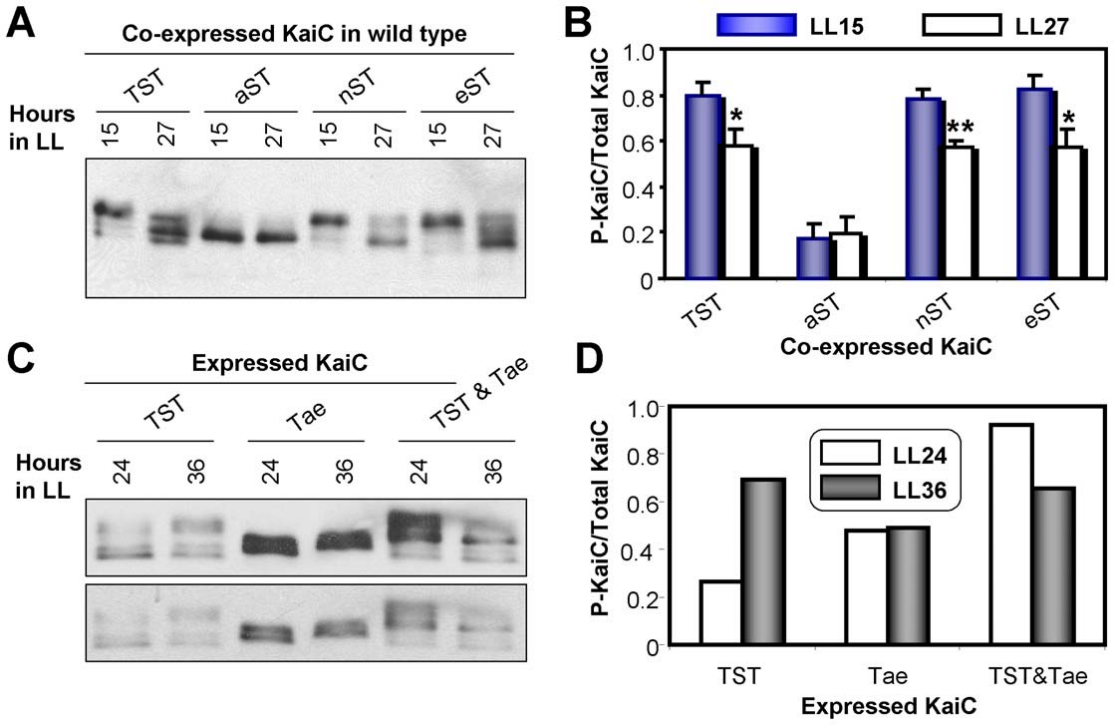
Effect of co-expression of KaiCs in the wild-type strain on the KaiC phosphorylation patterns *in vivo* at two phases. (***A***) Immunoblot analysis of KaiC in strains co-expressing KaiCs mutated at residue 426 with wild-type KaiC at 15 h or 27 h in LL: WT/TST, WT/aST, WT/nST, or WT/eST. (***B***) Ratios of the P-KaiC/total KaiC from densitometry of (*A*). *, *p*<0.05; **, *p*<0.01 (Student's t-test; error bars  =  SD). (***C***) KaiC expression profiles at 24 h or 36 h in LL in strains expressing wild-type KaiC^TST^ or the S431/T432 double mutant KaiC^Tae^ alone or co-expressing KaiC^Tae^ with wild-type KaiC^TST^ (TST & Tae). Lower panel is a shorter exposure of the blot to show the double bands of KaiC^Tae^ mobility. (***D***) Ratios of the P-KaiC:total KaiC from densitometry of panel *C*.

A possible trivial explanation for the data of Figure 2 might be that KaiC^TST^ or KaiC^aST^ are soluble and active when expressed intracellularly, but that KaiC^nST^, KaiC^eST^, and Kai^Tae^ aggregate and become inactive. However, this is not the case. All the versions of KaiC have a significant soluble pool when expressed *in vivo* (the proportion of soluble to insoluble KaiC is essentially the same for the mutants as for KaiC^TST^, Fig. S2). Also, all the mutant versions of KaiC have an activity *in vivo* because they either lengthen the period significantly or abolish rhythmicity altogether in the co-expression experiment (Fig. 2). Because rhythmic phosphorylation of KaiC is critical to the clock's operation [3], [17], [26], [27], perhaps the effect of the 426 site mutations can be explained by an effect on phosphorylation patterns. Figure 3 depicts the total KaiC phosphorylation patterns *in vivo* at two circadian phases in the co-expression experiment. There are significant day/night changes in phosphorylation of the total KaiC pool for coexpression of KaiC^TST^, KaiC^nST^, KaiC^eST^, and Kai^Tae^. On the other hand, the coexpression of KaiC^aST^ results in constitutive hypophosphorylation of the total KaiC pool (including the endogenous KaiC^TST^). Beyond these overall changes in phosphorylation levels, the patterns themselves are not additionally informative. Therefore, the phosphorylation patterns under the KaiC coexpression protocol *in vivo* are consistent with the data obtained by monitoring the luminescence rhythms (Fig. 2) in that the combinations that show a luminescence rhythm correlate with those that show day/night differences in phosphorylation patterns. The data of Figs. 1–3 support the conclusion that T426 serves as a crucial site for proper clock function despite the fact that the mass spectrometry approach did not indicate a participation of T426 in the phosphorylation sequence [26], [27].

### Phosphorylation *in vitro* of KaiC mutants at residue 426

**Figure 4:**
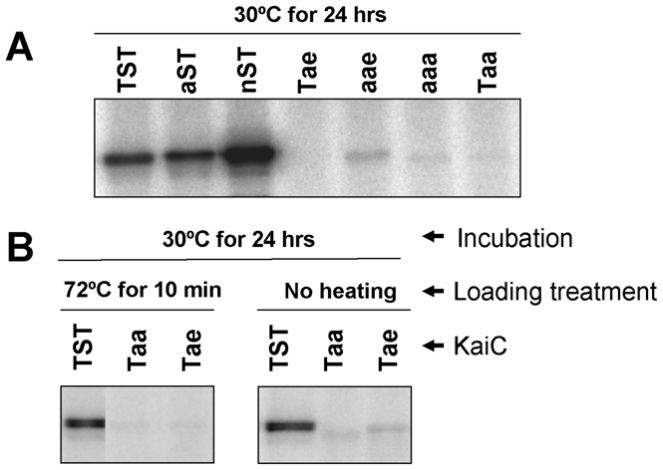
^32^P-labeled phosphorylation of wild-type or mutant KaiCs. Purified wild-type KaiC (TST) or mutant KaiCs (aST, nST, Tae, Taa, aae, or aaa) were incubated at 30°C for 24 hours in the reaction buffer containing [γ-^32^P]ATP. (***A***) Before loading, the samples were heated at 100°C. (***B***) Before loading, the samples were heated to 72°C (left portion of panel *B*) for 10 m, or loaded without heating above room temperature (right portion of panel *B*). Following SDS-PAGE, ^32^P-labeled KaiC was detected by autoradiography.

However, the crystallography results of the accompanying manuscript indicate that T426 can auto-phosphorylate in KaiC^Tae^ crystals, i.e., when 432 is activated by a glutamate and 431 is an alanine and therefore both site 431 and site 432 are unavailable for phosphorylation [28]. In an attempt to demonstrate under other conditions if T426 can be stably phosphorylated, we performed *in vitro* phosphorylation assays in the presence of ATP with a ^32^P radiolabel at the γ-phosphate position. As shown in Fig. 4A, KaiC^TST^, KaiC^aST^, and KaiC^nST^ robustly label with ^32^P at 30°C for 24 h presumably because S431 and T432 are available for phosphorylation in these proteins. The control proteins KaiC^aaa^ and KaiC^Taa^ show very low labeling with ^32^P, while the control protein KaiC^aae^ has labeling above background (presumably residues other than 426, 431, and 432 can sometimes phosphorylate). However, KaiC^Tae^ does not show labeling above background by this assay when the samples are heated before loading prior to SDS-PAGE (Fig. 4A and left panel of Fig. 4B). On the other hand, when the samples are not heated before loading on the SDS-PAGE gel, there is a low but significant labeling with the ^32^P radiolabel of the KaiC^Tae^ mutant protein that is slightly above the level in KaiC^Taa^ (Fig. 4B, right panel). The crystallographic results indicate that KaiC^Tae^ can be phosphorylated at T426 under specific conditions [28], but the results of Fig. 4 indicate that other conditions do not promote stable phosphorylation. Moreover, the comparison of results obtained when the samples were heated vs. not heated (Fig. 4B) prior to SDS-PAGE analysis suggest that phosphorylation on residues other than S431 and T432 (possibly at T426) is labile.

### Mutations at T426 affect KaiC dephosphorylation kinetics

**Figure 5:**
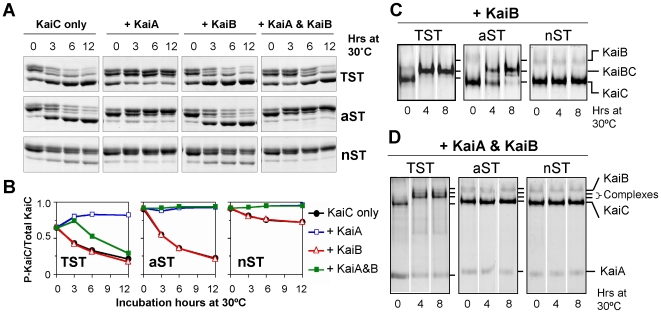
T426 mutations affect the rate of KaiC dephosphorylation and the formation of Kai complexes *in vitro*. (***A***) Dephosphorylation rate of wild-type KaiC (KaiC^TST^) and T426 mutant KaiCs (KaiC^aST^ and KaiC^nST^). Purified KaiC proteins in the hyperphosphorylated state were incubated at 30°C with or without KaiA and/or KaiB for up to 12 h. (***B***) Densitometry of the KaiC phosphorylation/dephosphorylation profiles from panel A. (***C***) & (***D***) Detection of Kai complexes. Purified KaiC^TST^, KaiC^aST^ or KaiC^nST^ were incubated with KaiB (panel C) or KaiA & KaiB (panel D) at 30°C for 8 h. The formation of KaiBC complex (panel *C*) and/or KaiBC/ABC complexes (panel *D*) were then identified by native gel analyses. Positions of KaiA, KaiB, KaiC and KaiBC/ABC complexes on native gel are noted.

Our original hypothesis for the role of T426 was that a phosphate could shuttle between T426 and S431, either as a stable phosphorylation or as a labile interaction [25]. The crystal structure of KaiC^Tae^ supports the hypothesis that T426 can auto-phosphorylate [28], while the data of Fig. 4B supports the hypothesis that the putative phosphate-T426 interaction is unstable. A different possible role for T426, however, is that this threonine acts to stabilize/modulate the phosphorylation on S431 and thereby regulates the phospho-S431 site that is key to the binding of KaiB and subsequent steps in the dephosphorylation sequence [26], [27]. Figure 5 provides data that support this alternative hypothesis. Figs. 5A and 5B show that KaiC^TST^ and KaiC^aST^ auto-dephosphorylate at approximately the same rates. Conversely, replacement of threonine with asparagine at 426 (i.e., KaiC^nST^) very significantly hinders the rate of dephosphorylation. KaiA stimulates the phosphorylation of all three forms of KaiC, while KaiB alone has little effect on the rate of dephosphorylation for either KaiC^TST^ or KaiC^aST^ (and KaiB has no effect on KaiC^nST^, which remains hyperphosphorylated). However, when all three Kai proteins are put together, there is a striking difference between KaiC^TST^ and KaiC^aST^. For KaiC^TST^, KaiB antagonizes KaiA's stimulation of phosphorylation and therefore KaiC^TST^ dephosphorylates as reported previously [20]. In the cases of KaiC^aST^ and KaiC^nST^, however, KaiB is incapable of antagonizing KaiA's effect and these versions hyperphosphorylate in the presence of KaiA and KaiB *in vitro* (Fig. 5A,B).

### Mutations at T426 affect formation of complexes between KaiC and KaiA/KaiB

It might be imagined that KaiB's inability to antagonize KaiA's action on KaiC^aST^ or KaiC^nST^ is because KaiB cannot bind these versions of KaiC. Fig. 5C shows that this could be the case for KaiC^nST^—by a native gel analysis, KaiC^nST^ does not form a stable complex with KaiB. However, the same analysis indicates that both KaiC^TST^ and KaiC^aST^ form stable complexes with KaiB (Fig. 5C; the KaiC^aST^/KaiB complex forms more slowly than the KaiC^TST^/KaiB complex). When mixed with both KaiA and KaiB, KaiC^TST^ forms stable ABC complexes, but neither KaiC^aST^ nor KaiC^nST^ can form stable complexes with KaiA and KaiB (Fig. 5D). Taken together, the data of Fig. 5 show that KaiB binds to both KaiC^TST^ and KaiC^aST^, but is incapable of antagonizing KaiA's hyperphosphorylation of the KaiC^aST^ mutant. KaiC^nST^ cannot bind KaiB in stable complexes and is deficient in its ability to auto-dephosphorylate. When both KaiA and KaiB are present, KaiC^aST^ and KaiC^nST^ are incapable of forming stable ABC complexes (Fig. 5D) and are unable to dephosphorylate normally (Fig. 5A & 4B). These data indicate that substitution of threonine at site 426 with non-phosphorylatible residues prevents normal interactions with KaiA and KaiB and also prevents the proper regulation of KaiC phosphorylation status.

Clearly, S431 and T432 are key phosphorylation sites on KaiC [19], [24]–[27]. While the crystallographic and mutational analyses support a crucial role for T426 [24], [25], e.g., as a potential additional phosphorylation site, mass spectrometry measurements did not detect a stable phosphorylation at the T426 residue [26], [27]. However, if phosphorylation at T426 is labile, the phosphate group might have been lost during preparation of the proteins for mass spectrometry. Further crystallographic analyses support the earlier interpretation that T426 can be phosphorylated [28], although the data shown in Fig. 5D indicates that phosphorylation in the KaiC^Tae^ mutant (possibly at T426) may be an unstable modification. Substitutions at 426 with either alanine or asparagine nonetheless show *in vitro* that T426 is crucial to the action of KaiB, either its binding (asparagine substitution) or its activity once bound (alanine substitution). *In vivo*, substitution of threonine with alanine at 426 shows that rhythmicity is abolished when the mutant KaiC is expressed alone (Fig. 1) and disrupts the *in vivo* rhythm when the mutant KaiCs are co-expressed with wild-type KaiC^TST^ (Fig. 2). Therefore, we demonstrate here that 426 is a crucial site for the regulation of KaiC phosphorylation status and that a threonine residue at this site is essential for proper clock function. Our findings strongly support that these KaiC phosphorylation sites coordinately regulate key circadian properties in cyanobacteria, including period, amplitude, robustness, and temperature compensation.

## Supporting Information

Table S1 Synechococcus strains used in this study. “Strain” is the name of the strain based on the residues at the 426, 431, and 432 positions and the promoter used. “Description” is the full description of the strain. “Reporter” is the promoter used in the luxAB fusion and the neutral site in which the reporter is inserted. NSI  =  neutral site I, NSII  =  neutral site II. Antibiotic resistances: Spr  =  spectinomycin, Cmr  =  chloramphenicol, Kmr  =  kanamycin.
(0.03 MB DOC)

Figure S1 KaiC phosphorylation status is cyclic in vivo in the kaiC-null strain expressing wild-type KaiC-TST, but it is not cyclic when mutant KaiCs (KaiC-aST, KaiC-TaT, KaiC-TSa, KaiC-aaT, or KaiC-aaa) are expressed. For expression of a single-copy of the kaiC gene in cyanobacteria in the experiment depicted in this figure, the trc promoter-driven wild-type or mutant kaiC gene was introduced into neutral site II (NS II) of an in-frame kaiC-deletion strain (for references, see: Ditty et al. 2005 Microbiology 151: 2605–2613 and Xu et al. 2003 EMBO J 22: 2117–2126). This strain also includes the kaiBCp::luxAB reporter. After two LD 12:12 cycles, the cultures were released to constant light (LL), and the cells were harvested every 6 h in LL. The extracts were subjected to the KaiC immunoblot assay. The position of hyper-phosphorylated KaiC and hypo-phosphorylated KaiC are marked by solid and open arrows, respectively.
(1.18 MB TIF)

Figure S2 Comparison of KaiC expression between soluble and insoluble portions from the wild type strain co-expressing wild-type or mutant KaiCs. After 12 h darkness, the cultures of the wild-type strain co-expressing trcp-driven wild-type KaiC (KaiC-TST) or mutant KaiCs (as indicated) were released to LL, and 0.1 mM of IPTG was added. The cells were incubated for 6 h (from LL0 to LL6) before harvesting. The soluble extracts (upper panel), made in the SDS-free buffer, and the insoluble extracts (lower panel), made from the pellets in the SDS-containing buffer, were subjected to immunoblot analysis.
(0.41 MB TIF)
