# Supplementary material for: Intramolecular Regulation of Phosphorylation Status of the Circadian Clock Protein KaiC
Source: PLoS One. 2009 Nov 25;4(11):e7509. doi: 10.1371/journal.pone.0007509 (PMC2778140; doi:10.1371/journal.pone.0007509)
Supplement: Table S1 — Synechococcus strains used in this study. “Strain” is the name of the strain based on the residues at the 426, 431, and 432 positions and the promoter used. “Description” is the full description of the strain. “Reporter” is the promoter used in the luxAB fusion and the neutral site in which the reporter is inserted. NSI = neutral site I, NSII = neutral site II. Antibiotic resistances: Spr = spectinomycin, Cmr = chloramphenicol, Kmr = kanamycin. (0.03 MB DOC) [file pone.0007509.s001.doc]

**Table S1.** *Synechococcus* strains used in this study

**Strain Description Reporter**

WT Wild-type *Synechococcus* (wild-type KaiC^WT^ = KaiC^TST^) *psbAI*p::luxAB (Sp^r^) in NS I

TST KaiABC^TST^ (i.e. KaiC^WT^; Sp^r^) in *kaiABC*-null strain *kaiBC*p::luxAB (Cm^r^) in NS I

aST KaiABC^aST^ (i.e. KaiC^T426A^; Sp^r^) in *kaiABC*-null strain *kaiBC*p::luxAB (Cm^r^) in NS I

nST KaiABC^nST^ (i.e. KaiC^T426N^; Sp^r^) in *kaiABC*-null strain *kaiBC*p::luxAB (Cm^r^) in NS I

eST KaiABC^eST^ (i.e. KaiC^T426E^; Sp^r^) in *kaiABC*-null strain *kaiBC*p::luxAB (Cm^r^) in NS I

Tae KaiABC^Tae^ (i.e. KaiC^S431A/T432E^; Sp^r^) in *kaiABC*-null strain *kaiBC*p::luxAB (Cm^r^) in NS I

Tee KaiABC^Tee^ (i.e. KaiC^S431E/T432E^; Sp^r^) in *kaiABC*-null strain *kaiBC*p::luxAB (Cm^r^) in NS I

trc-TST *trc*p::KaiC^TST^ (i.e. KaiC^WT^; Km^r^) in *kaiC*-null strain’s NS II *kaiBC*p::luxAB (Sp^r^) in NS I

trc-aST *trc*p::KaiC^aST^ (i.e. KaiC^T426A^; Km^r^) in *kaiC*-null strain’s NS II *kaiBC*p::luxAB (Sp^r^) in NS I

trc-TaT *trc*p::KaiC^TaT^ (i.e. KaiC^S431A^; Km^r^) in *kaiC*-null strain’s NS II *kaiBC*p::luxAB (Sp^r^) in NS I

trc-TSa *trc*p::KaiC^TSa^ (i.e. KaiC^T432A^; Km^r^) in *kaiC*-null strain’s NS II *kaiBC*p::luxAB (Sp^r^) in NS I

trc-aaT *trc*p::KaiC^aaT^ (i.e. KaiC^T426A/S431A^; Km^r^) in *kaiC*-null strain’s NS II *kaiBC*p::luxAB (Sp^r^) in NS I

trc-aaa *trc*p::KaiC^aaa^ (i.e. KaiC^T426A/S431A/T432A^; Km^r^) in *kaiC*-null strain’ NS II *kaiBC*p::luxAB (Sp^r^) in NS I

WT/TST WT strain coexpressing *trc*p::KaiC^TST^ (i.e. KaiC^WT^; Km^r^) in NS II *psbAI*p::luxAB (Sp^r^) in NS I

WT/aST WT strain coexpressing *trc*p::KaiC^aST^ (i.e. KaiC^T426A^; Km^r^) in NS II *psbAI*p::luxAB (Sp^r^) in NS I

WT/nST WT strain coexpressing *trc*p::KaiC^nST^ (i.e. KaiC^T426N^; Km^r^) in NS II *psbAI*p::luxAB (Sp^r^) in NS I

WT/eST WT strain coexpressing *trc*p::KaiC^eST^ (i.e. KaiC^T426E^; Km^r^) in NS II *psbAI*p::luxAB (Sp^r^) in NS I

WT/Tae WT strain coexpressing *trc*p::KaiC^Tae^ (i.e. KaiC^S431A/T432E^; Km^r^) in NS II *psbAI*p::luxAB (Sp^r^) in NS I
