# Supplementary figures and images for: Intramolecular Regulation of Phosphorylation Status of the Circadian Clock Protein KaiC
Source: PLoS One. 2009 Nov 25;4(11):e7509. doi: 10.1371/journal.pone.0007509 (PMC2778140; doi:10.1371/journal.pone.0007509)

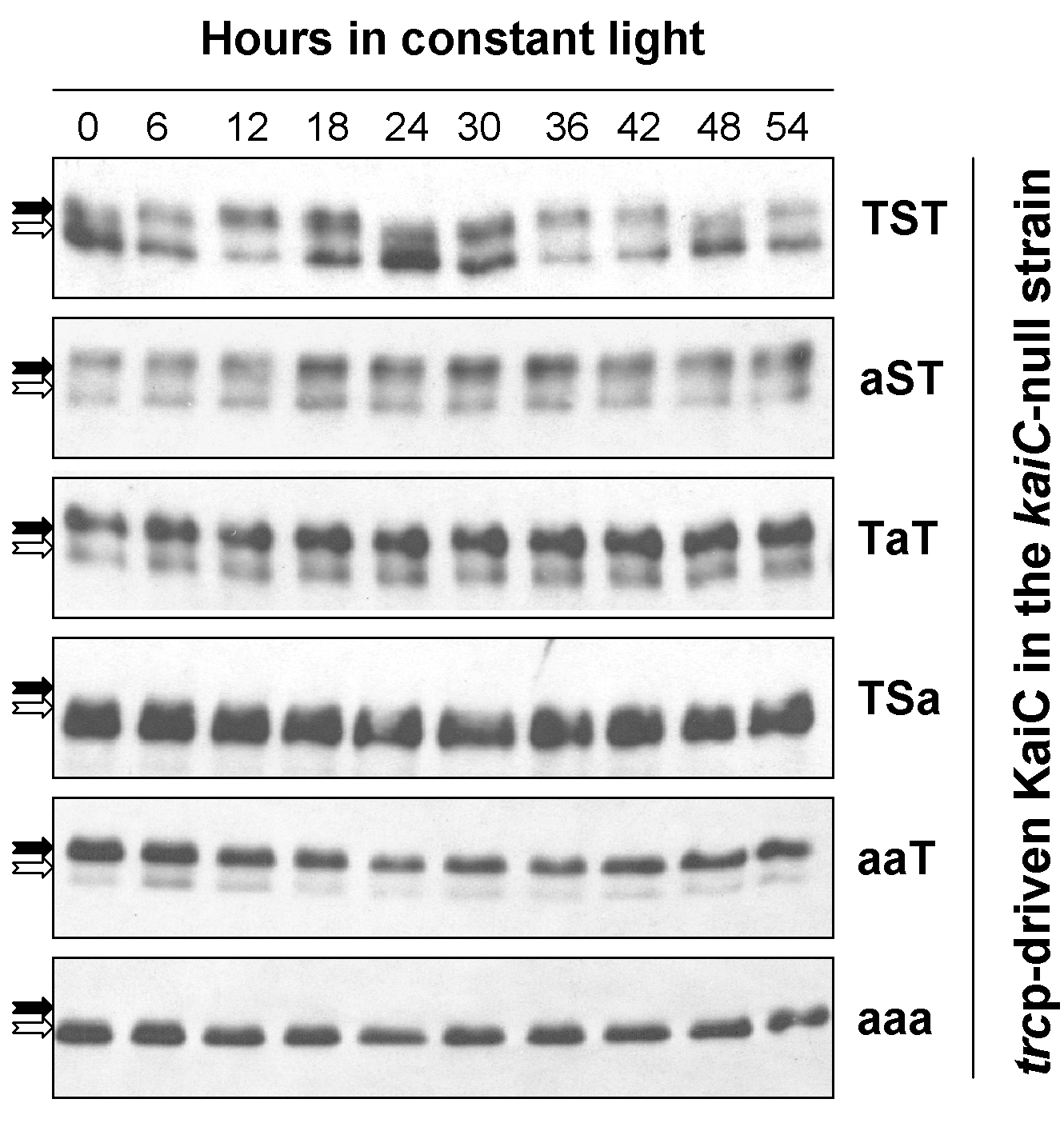

Supplement: Figure S1 — KaiC phosphorylation status is cyclic in vivo in the kaiC-null strain expressing wild-type KaiC-TST, but it is not cyclic when mutant KaiCs (KaiC-aST, KaiC-TaT, KaiC-TSa, KaiC-aaT, or KaiC-aaa) are expressed. For expression of a single-copy of the kaiC gene in cyanobacteria in the experiment depicted in this figure, the trc promoter-driven wild-type or mutant kaiC gene was introduced into neutral site II (NS II) of an in-frame kaiC-deletion strain (for references, see: Ditty et al. 2005 Microbiology 151: 2605–2613 and Xu et al. 2003 EMBO J 22: 2117–2126). This strain also includes the kaiBCp::luxAB reporter. After two LD 12:12 cycles, the cultures were released to constant light (LL), and the cells were harvested every 6 h in LL. The extracts were subjected to the KaiC immunoblot assay. The position of hyper-phosphorylated KaiC and hypo-phosphorylated KaiC are marked by solid and open arrows, respectively. (1.18 MB TIF) [file pone.0007509.s002.tif]

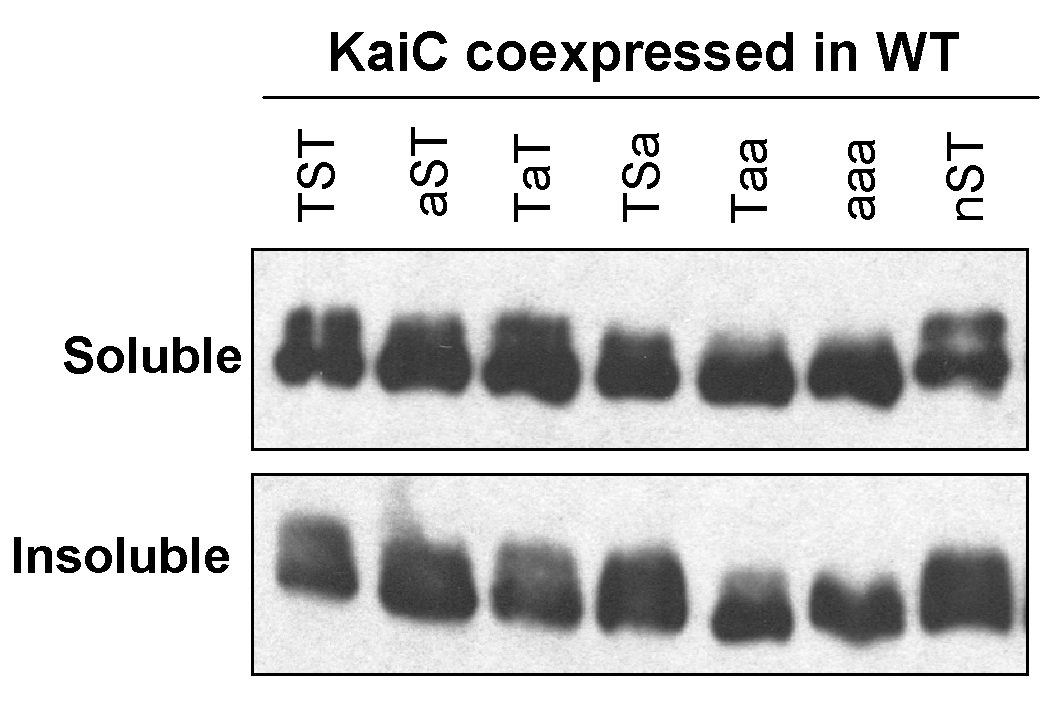

Supplement: Figure S2 — Comparison of KaiC expression between soluble and insoluble portions from the wild type strain co-expressing wild-type or mutant KaiCs. After 12 h darkness, the cultures of the wild-type strain co-expressing trcp-driven wild-type KaiC (KaiC-TST) or mutant KaiCs (as indicated) were released to LL, and 0.1 mM of IPTG was added. The cells were incubated for 6 h (from LL0 to LL6) before harvesting. The soluble extracts (upper panel), made in the SDS-free buffer, and the insoluble extracts (lower panel), made from the pellets in the SDS-containing buffer, were subjected to immunoblot analysis. (0.41 MB TIF) [file pone.0007509.s003.tif]
